# Opportunities to use electronic health record audit logs to improve cancer care

**DOI:** 10.1002/cam4.4690

**Published:** 2022-03-29

**Authors:** Yash S. Huilgol, Julia Adler‐Milstein, Susan L. Ivey, Julian C. Hong

**Affiliations:** ^1^ UC Berkeley‐UCSF Joint Medical Program University of California Berkeley California USA; ^2^ School of Medicine University of California San Francisco California USA; ^3^ Center for Clinical Informatics and Improvement Research (CLIIR) University of California San Francisco California USA; ^4^ School of Public Health University of California Berkeley California USA; ^5^ Bakar Computational Health Sciences Institute University of California San Francisco California USA; ^6^ Department of Radiation Oncology University of California San Francisco California USA

**Keywords:** audit log, decision‐making, digital health, electronic health records, informatics, quality and safety

## Abstract

The rapid adoption of electronic health records (EHRs) has created extensive repositories of digitized data that can be used to inform improvements in care delivery, processes, and patient outcomes. While the clinical data captured in EHRs are widely used for such efforts, EHRs also capture audit log data that reflect how users interact with the EHR to deliver care. Automatically collected audit log data provide a unique opportunity for new insights into EHR user behavior and decision‐making processes. Here, we provide an overview of audit log data and examples that could be used to improve oncology care and outcomes in four domains: diagnostic reasoning and consumption, care team collaboration and communication, patient outcomes and experience, and provider burnout/fatigue. This data source could identify gaps in performance and care, physician uptake of EHR features that enhance decision‐making, and integration of data trends for oncology. Ensuring researchers and oncologists are familiar with the data's potential and developing the data engineering capacity to utilize this rich data source, will expand the breadth of research to improve cancer care.

## Graphical abstract

This review discusses the current literature base for audit log research, which automatically captures electronic health record (EHR) user behavior and interactions with patient records. We place this work in context and propose questions to guide future oncology research to improve care decisions, quality, and outcomes.

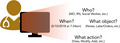

## 1 INTRODUCTION

The electronic health record (EHR) contains readily available, highly detailed data, which has the potential to be used to generate clinical insights.^1^, ^2^ Current cancer informatics research has focused on the clinical patient information contained within EHR.^3^, ^4^, ^5^ While these data are clinically useful, automatically collected, unprocessed data are available to analyze for research and quality improvement purposes that have been underutilized. We introduce the reader to the methodologies of using the audit log dataset in the field of oncology. As domain experts, oncologists can play a role in guiding the development and use of this methodology with health administrators and informatics researchers. This review can facilitate collaboration between oncologists, health administrators, and data scientists to better understand clinical EHR behaviors, and accordingly improve the EHR and their patient care.

### 1.1 Defining the audit log dataset

**FIGURE 1:**
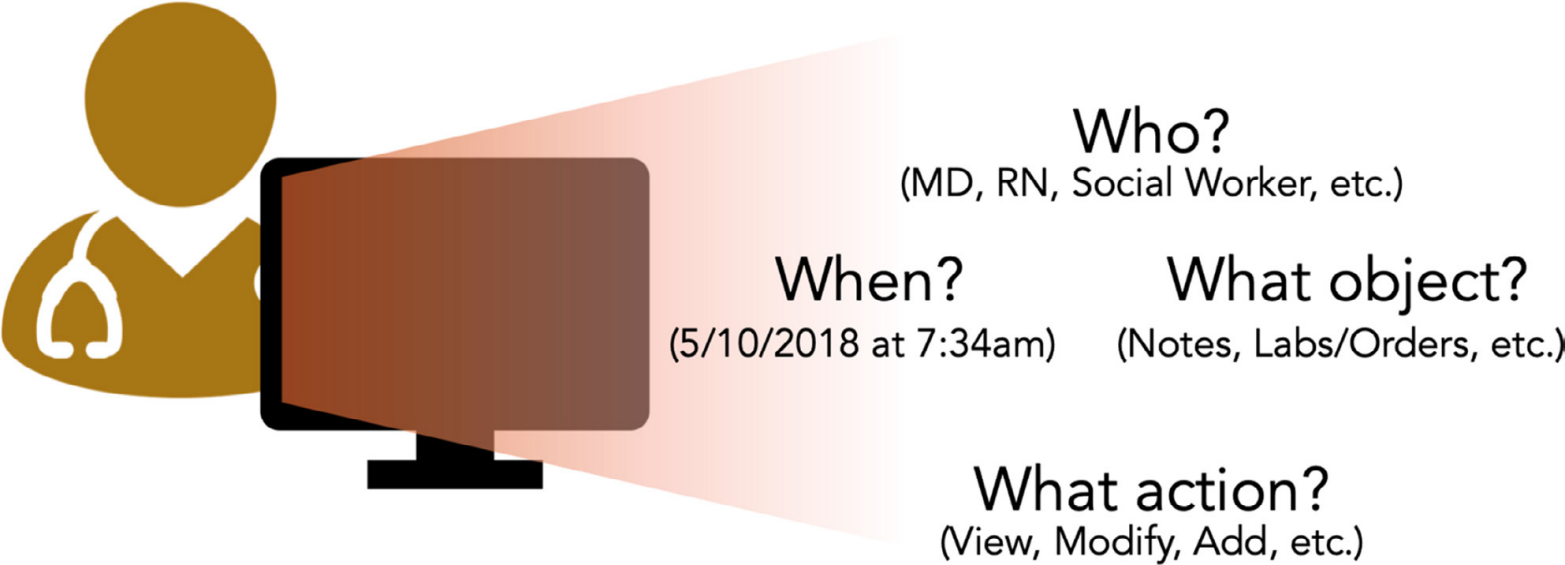
Automatic data collection in the audit log dataset. The information in this figure is collected automatically as part of the audit log metadata. While initially collected solely for security reasons, this data can be repurposed for informatics and health services research into EHR user behavior

The audit log is defined as the metadata and details of every user interaction within the EHR. This information is automatically collected by the EHR system. Historically, these data have been used to audit any security breaches to key information technology (IT) infrastructure. Figure 1 shows how the audit log tracks information about different aspects of patient record access: the viewer, the action, the timestamp, and the patient record.^6^

## 2 THE AUDIT LOG IN ONCOLOGY

Health information technology in the past two decades has enabled this highly detailed, audit log data to be collected. The Health Information Technology for Economic and Clinical Health (HITECH) Act in 2009 led to the broad adoption of electronic health records, which has been ubiquitously used by clinicians. Anticipating the importance of privacy protections in this software, the EHR audit log was required to be collected as part of the Health Insurance Portability and Accountability Act (HIPAA) Security Rule of 2005.^7^

In contrast with research conducted on clinical data entered by a health care professional into the EHR, audit log research is automatically derived from user behaviors. The audit log allows researchers to study physician behaviors, because it systematically captures data that reflects a user's behavior in a software system.^8^ The benefit of using the audit log for data collection, aggregation, and analysis is that there is no independent human observer necessary to conduct the collection.^8^ However, as introduced later in this review, the drawback to this data type is that clinicians and researchers are needed to interpret the audit log data elements and verify that the conclusions reflect real‐world behaviors. The gold standard for validation through manual data collected is by administering observation‐based time and motion studies, which have in the past been used to validate the audit log dataset and their behaviors.^9^, ^10^

### 2.1 A non‐traditional dataset for research

EHR audit log data represent a class of data that has rarely been used in research yet holds potential insights for cancer care improvement. Specifically, they can extend current research by better elucidating clinical decision‐making and health care processes that contribute to the quality and health services improvement missions of cancer care. This underutilized dataset accessible to most organizations using EHRs has the potential to fuel an integrated learning ecosystem between clinical practice and research.^5^, ^11^ Many audit log datasets can also be used in conjunction with other user‐level, time‐level, or patient‐level datasets and variables that make it a versatile tool to collect insights about health services research.

## 3 OPPORTUNITIES FOR RESEARCH

The use of the audit log in oncology is limited. We characterize themes by research questions, identifying four possible oncology research areas that could be advanced via using EHR audit log data: diagnostic reasoning and consumption, care team collaboration and communication, patient outcomes and experience, and EHR user characterizations. For those seeking a more technical review, Rule et al.^6^ provide a list of previous work organized by technique and specialty.

### 3.1 Diagnostic reasoning and consumption

It is cognitively challenging for oncologists to find and consume multiple streams of data that are available in the EHR (radiology, pathology, labs, and notes) in an efficient manner.^12^ Current EHRs have been designed for coding and structuring forms, rather than for streamlining doctor or caregiver use to make and interpret decisions.^13^, ^14^ While chart review of historical data is important, many clinicians worry about overlooking relevant information due to the volume of data and differing EHR configurations.

The audit log can assist in tracking a user's information consumption pattern, which can be a proxy for a physician's decision‐making. Audit log studies can be useful to confirm self‐reported survey data collected about EHR review by physicians. A study found that while nearly all intensive care unit (ICU) clinicians surveyed perform an EHR review when admitting new patients, nearly half (49%) of clinicians indicated that a significant amount of time was spent reviewing EHR charts “haphazardly,” with searches going back three or more years to find relevant information.^12^ Information‐seeking behaviors also differed.^15^

Prior studies have studied how physicians might consume or add information. A study of task analyses of 16 physicians tried to understand what relevant notes are reviewed for particular appointments, given the cognitive burden of current charts, for both acute and chronic clinic visit types.^16^ Note review and input method varied by specialty: specialists tended to prefer using a voice recorder, while primary care physicians tended to use templates tailored for visit types (e.g., “well visit” vs. urgent problem‐based visit).^17^ Consumption can also include information seeking and charting. Commonly searched terms can be used to improve the EHR's ability to conveniently retrieve this information and train providers more effectively.^18^ The study was also able to determine that most physicians used the search feature, but its use was generally low among nonphysicians and pharmacists.

Given the cognitive burden of excessive information, access log studies can also be used to predict EHR user roles based on their behavior, and assess if particular roles are given the access appropriate for their level and usage.^19^, ^20^ This is important because if data are not necessary for particular roles to access, its presence may overwhelm the user and prevent them from properly performing their tasks.

These studies have implications for cancer care since audit log studies could identify optimal review strategies to improve workflows and make it more efficient for physicians to find relevant information in large patient files. These could also impact the design and development of new features in the EHR to make it easier to find the information that is needed specifically for cancer patients.

### 3.2 Care team collaboration and communication

Cancer care is an inherently collaborative and interdisciplinary effort, with many different care providers providing distinct support to cancer patients. In the literature, high intensity of interprofessional collaboration is associated with increased patient satisfaction, which is associated with improved care, cost control, and reduction in clinical errors.^21^, ^22^ Cancer care often is centered around multidisciplinary patient tumor boards or team huddles to review patient records. Improving interprofessional, team‐based performance can potentially improve care quality, impact patient diagnoses and treatments, and outcomes.^23^, ^24^, ^25^, ^26^, ^27^ However, understanding how different members of a care team might interact with one another–especially through an EHR–is presently a complex undertaking. For example, each team member may interact with notes asynchronously or access different information panels. It is also not known whether certain team interactions may occur only in an EHR, and not in‐person, which is how collaboration is conventionally defined.

Assessing team‐based collaborations and the logistics of care administration is a possible use case for audit log data. For example, audit log studies to understand collaboration and communication have been conducted in obstetrics/gynecology and neonatal care.^28^, ^29^ Audit log studies outside of the oncology context have found that as team size increased, so too did the use of electronic actions, daily hours of use, and the number of computer sessions.^30^ This means that larger teams are associated with increased interactions with the EHR. Another study found that greater delegation of tasks within clinician teams is associated with improved productivity, as measured by work relative value units (RVUs).^31^ Studies have also been conducted to determine which members of the care team collaborate at different times in the patient's visit cycle.

Of the domains discussed in this paper, collaboration and communication have been the most robustly studied in the oncology context using the audit log. Some examples include network analyses to better understand the different physicians who contributed notes to patient encounters. A study of surgical colorectal cancer audit log records found that a given health care professional was on average connected with six other professionals for each patient record.^32^ These studies can assess not only who is on a particular patient team, but also to what extent each team member plays a role. These data could also be used to understand whether certain principal care team members share increased responsibilities in communicating with one another and interacting on the patient record.^33^ Future research might consider which roles tend to have higher EHR use burden, and identify areas in which communication and interfaces are lacking, especially for patient supportive care services and social work. If these deficiencies exist, these studies could allow programs to prioritize cross‐training and familiarity between roles to ensure linkages and continuity of care. These audit log studies can determine if collaboration on patient records occurs at a given point in time.^34^, ^35^ These studies could complement prior research that attempts to understand the order in which events take place when providing clinical pathways for treatment.^36^

### 3.3 Patient outcomes and experience

Oncology is characterized as a long‐term journey with the potential for many touchpoints with the medical system. Further research is necessary to study patterns in EHR use and downstream outcomes, such as patient outcomes (e.g., emergency care events) or patient‐reported outcomes (e.g., satisfaction). Though the literature is sparse, we describe how work in other care contexts could guide research in the oncology context.

These studies could be conducted in an inpatient environment specifically studying those who care for hospitalized oncology patients. For example, a study conducted in a general medicine ward found an association between EHR‐measured work hours and a patient outcome composite variable of mortality, transfer to the ICU, or 30‐day same‐hospital readmission.^30^ This study confirmed previous literature which suggested that high workload (including EHR use burden) can lead to worse patient outcomes, such as increased length of stay and ICU transfers.^37^, ^38^

The audit log literature has found contradictory effects on the role of EHR usage and patient outcomes, and likely is specific to the ambulatory context. For the emergency department, these could include door‐to‐disposition, throughput efficiency, or readmissions. For example, a study of physician EHR usage activities in the emergency department found that there were positive correlations between physician EHR review time and door‐to‐disposition.^39^ In contrast, overall emergency department throughput efficiency in the emergency department decreased with increased time for physician's note review.^39^ Another study found that review of notes by an emergency physician led to a decrease in 7‐day readmission by 6%.^40^ This work would continue efforts underway in the outpatient cancer care context, where researchers have already started studying oncology treatment‐based acute care needs or unplanned care visits.^41^, ^42^, ^43^

Another example of an outpatient oncology study could be studying EHR use and patient satisfaction. A pilot study of general internists and medical subspecialists' daytime EHR usage was inversely related to patient satisfaction metrics.^44^ Audit log data could also be used to study the efficacy and patient satisfaction metrics of those physicians who are early adopters of new EHR features, such as an interoperable integrated view.^45^ These types of studies could be conducted in an outpatient oncology context and study informatics initiatives designed to improve patient care.

Ultimately, further research is needed to disentangle whether patient outcomes or patient‐reported outcomes are impacted positively or negatively by a physician's EHR usage. In oncology, it is not yet known if the audit log could provide a useful dataset for further investigation of patient outcomes or patient experience.

### 3.4 EHR use and burnout

The causes of burnout are multifaceted and well documented in oncology.^46^, ^47^, ^48^ Burnout and emotional fatigue can affect individuals differently, but one way to study these differences is through EHR use patterns. For instance, clinicians at an academic medical center spent about one‐third of their EHR usage after hours.^49^ Workload and task demand could heterogeneously impact specific members of the cancer care team. In this section, we describe how differing EHR user behavior could have implications for burnout.

Audit log studies have found differences in EHR behavior by gender. In an ambulatory setting, female physicians spent more time than male physicians in the EHR on weekends and after hours.^50^ Second, audit log studies to study physician EHR usage have been conducted across specialties. General surgery residents spent more time per patient than orthopedic surgery residents on EHR time, chart review, and documentation.^51^ EHR use behaviors were also dependent on the service through which residents were rotating and the attending on each team.^52^, ^53^ Prior studies have considered a diverse number of specialties (e.g., radiation oncology, dermatology). Often, these papers do not provide disaggregated analyses on these groups.^54^

Future research could disaggregate EHR use burden and present a complement to published literature on burnout and charting burden in oncology. For example, one paper found that surgical oncologists spent the most time using the EHR compared to other surgeons with a mean usage time of 2.5 h. When disaggregated, breast surgeons' use was even higher than other surgical oncologists.^55^

The research priorities are to focus on different cancer specialties and training levels within each (e.g., internal medicine residents and fellows vs. radiation oncology residents and fellows) to better understand their behaviors. A next step might be to consider how these behaviors might be correlated with burnout and job satisfaction specifically in the oncology context. Furthermore, additional studies are needed to better understand the causal pathway of EHR use and burnout, in consideration of other variables known to contribute to burnout. For example, one recent study found that adjusting by clinician sex and work culture play a more significant role in predicting burnout than EHR use.^56^

Though differences in EHR usage are not inherently good or bad, they could play a role in explaining burnout on an oncology care team. Identifying those users' behaviors and characteristics (e.g., if they spend excessive time on EHRs after hours or bear a disproportionate burden) could be useful to improve the health and well‐being of physicians.^47^, ^57^, ^58^ These studies could be correlated with job satisfaction data and burnout, and help inform institutional initiatives and policy.

## 4 THE CLINICAL ONCOLOGIST'S ROLE

Due to its implications on practice, domain experts will play an indispensable role in the cycle of audit log research, from conception to EHR redesign, based on findings in these data.

### 4.1 Project conception

Informaticians and health system administrators often lack the context to understand how to improve the physician experience in the EHR. The oncologist in audit log research could collaborate with these individuals to propose appropriate research questions (e.g., with the domains mentioned above) or tie usage to EHR user behaviors or meaningful patient outcomes. The sheer volume of data being automatically collected needs to be better leveraged to improve care, so oncologists could propose to repurpose the data to answer pressing clinical questions.

### 4.2 Data validation

The audit log dataset will also need to be properly validated, as the use of these data to answer clinical questions remains in its nascence. For example, the EHR log may consider a window of time that either overestimates (e.g., physician steps away from EHR to see patient with the session open) or underestimates (e.g., physician has multiple windows or panels open at the same time) user actions.

These unique differences also may contribute to difficulty generalizing the data across different institutions or clinical sites with different EHR features. Therefore, it is in the interests of oncologists and those curating these data to ensure that physician EHR use is accurate. Oncologists can collaborate with researchers in in‐person observational studies collected in time and motion studies, which have previously research studies that have been conducted with EHR audit logs.^9^, ^10^

### 4.3 Contextualizing findings with other research spheres

As leaders in their clinical environments, oncologists can collaborate with informaticians and health administrators to contextualize the findings of audit log studies in their daily clinical environments and lead to quality improvement. For example, research has also found that the EHR burden is also disproportionately impacting women and younger trainees, so experts and clinical leaders can better contextualize and understand where disparities in their care team's EHR usage could be occurring. Oncologists can also ensure synergies with current precision medicine and implementation sciences efforts in cancer care. Ensuring synergy could support effective practices.^2^

### 4.4 EHR redesign

Beyond providing qualitative data to EHR developers on how best they could redesign EHR infrastructure, oncologist‐researchers can propose to test hypotheses on the most effective ways of navigating the EHR or facilitating searches. These findings, if proven quantitatively, could perhaps lead to changes in future EHR systems for their health system. By participating in research on audit log usage, oncologists could help to redesign the EHR to reduce their clicks or overall time in the EHR.

## 5 FUTURE OPPORTUNITIES

The study of the audit log, especially in oncology, is in its infancy. While the EHR audit log dataset has been studied in various contexts as described in the four domains, there are opportunities to tie this work more closely to the cancer context. While treatment and systems‐level interventions appear most obviously at the top of the cancer research agenda, there remains a substantial research gap in understanding the role of physician decision‐making in health and health care outcomes. Some considerations have already been studied elsewhere, including the use of heuristics, patient/physician decision‐making models, and time spent by physicians with patients.^56^, ^59^ The current cancer research agenda can be enhanced by considering the role that physician behavior might play in contributing to the quality of care, behavioral and gender care disparities, and physician/patient well‐being and clinical outcomes.

Any work conducted in the oncology community specifically would be part of a burgeoning literature base studying audit log usage. Utilizing the audit log would enable researchers to study how oncologists access relevant information in the EHR to make their treatment decisions, enhance team‐based collaboration, and improve patient outcomes in the process.

## CONFLICT OF INTEREST

Julia Adler‐Milstein is on the Board and holds shares in Project Connect that are unrelated to the current work. Julian Hong reported a patent for systems and methods for predicting acute care visits during outpatient cancer therapy pending that is unrelated to the current work. Yash S. Huilgol and Susan L. Ivey have no conflict to disclose.

## AUTHOR CONTRIBUTION

Conceptualization (YSH, JCH), methodology and design (YSH, JCH), supervision (JAM, SLI, and JCH), writing – original draft preparation (YSH), writing – review & editing (all authors).

## ETHICS STATEMENT

This review does not meet the criteria for human subject research by UC Berkeley Committee for Protection of Human Subjects and did not require ethics approval.

## Data Availability

Data sharing not applicable ‐ no new data generated, or the article describes entirely theoretical research.
